# Determining Which Hydrostatic Pressure Regimes Promote Osteogenesis in Human Mesenchymal Stem Cells

**DOI:** 10.1007/s13770-024-00666-w

**Published:** 2024-08-27

**Authors:** James R. Henstock, Joshua C. F. A. Price, Alicia J. El Haj

**Affiliations:** 1 Department of Applied Sciences, Northumbria University, Newcastle-upon-Tyne, NE2 1XE UK; 2 School of Pharmacy and Bioengineering, Guy Hilton Research Centre, Keele University, Stoke-on-Trent, ST4 7QB UK; 3 Healthcare Technologies Institute, School of Chemical Engineering, University of Birmingham, Birmingham, B15 2TH UK

**Keywords:** Bioreactor, Hydrostatic pressure, Mesenchymal stem cells, Mechanotransduction, Osteogenesis

## Abstract

**Background:**

Compressive loading of bone causes hydrostatic pressure changes which have been proposed as an osteogenic differentiation stimulus for mesenchymal stem cells (hMSCs). We hypothesised that hMSCs are adapted to differentiate only in response to cyclic hydrostatic pressures above critical thresholds of magnitude and frequency which correspond to physiological levels of anabolic bone loading.

**Methods:**

Using a pneumatic-hydrostatic bioreactor, we applied hydrostatic pressure regimes to human hMSCs in 3D collagen hydrogel cultures for 1 h/day over 28 days to determine which levels of pressure and frequency stimulated osteogenesis *in vitro*.

**Results:**

Stimulation of the 3D cultures with 0–280 kPa cyclic hydrostatic pressure at 1 Hz resulted in up to 75% mineralisation in the hydrogel (without exogenous growth factors), whilst static culture or variations of the regime with either constant high pressure (280 kPa, 0 Hz), low-frequency (0.05 Hz, 280 kPa) or low-magnitude (70 kPa, 1 Hz) stimulation had no osteogenic effects (< 2% mineralisation). Nuclear translocation of YAP was observed following cyclic hydrostatic pressure in mature MLO-A5 osteoblasts but not in hMSCs, suggesting that cyclic hydrostatic pressure activates different mechanotransduction pathways in undifferentiated stem cells and committed osteoblasts.

**Conclusions:**

Hydrostatic pressure is a potent stimulus for differentiating MSC into highly active osteoblasts and may therefore be a versatile tool for translational cell engineering. We have demonstrated that there are minimum levels of force and frequency needed to trigger osteogenesis, i.e. a pressure ‘switch’, which corresponds to the physiological forces experienced by cells in their native mesenchymal niche. The mechanotransduction mechanisms underpinning these effects are the subject of further study.

**Supplementary Information:**

The online version contains supplementary material available at 10.1007/s13770-024-00666-w.

## Introduction

We previously reported our development of a bioreactor which used compressed incubator air to generate dynamic hydrostatic pressures *in vitro* [1–3]. Hydrostatic (compressive) and hydrodynamic (streaming) forces are generated *in vivo* by compressive loads acting on interstitial fluids and are thought to be a principal way in which cells in bone sense loading and adapt the tissue to changing physiological demands [4–7].

The range of hydrostatic pressures which elicit osteogenic responses from cells in bone is therefore of interest, and from a bioengineering perspective presents a valuable tool for controlling stem cell differentiation through a user-directed engineering approach which can synergistically enhance or even replace growth factors as agents of differentiation [8, 9]. Precise control of *in vitro* forces during culture is now possible using bioreactors custom designed for mechanobiology research, and determining the range of forces which elicit particular responses from cells would help unlock the translation of mechanobiology into a new generation of manufacturing strategies for regenerative cell therapies and engineered tissues [10–12].

Considerable attention is also being paid to the mechanotransduction mechanisms which enable cells to incorporate mechanical stimuli from their environment into their biochemical signalling pathways, such as MAPK, Wnt and Hippo [13–15]. There is an ambition in the field to determine particularly dominant pathways which can serve as drug targets or biomarkers under defined conditions, and the Hippo pathway is a leading candidate for research in this area [16].

The Hippo signalling cascade, which involves intracellular transport of YAP/PDZ binding motif (TAZ) proteins has emerged as an important regulator of osteogenic differentiation resulting from changes in the mechanical environment of the extracellular matrix [17]. The current body of literature seems to indicate a complex, but nonetheless prominent role of YAP/TAZ in mechanosensing, suggesting that YAP/TAZ signalling is sensitive to both cell phenotype and the nature of stimulus applied (substrate stiffness, hydrodynamic shear, etc.) [18, 19].

We therefore set out to investigate the relative osteogenic potential of a series of hydrostatic pressure regimes on the differentiation and maturation of human mesenchymal stem cells (hMSCs) over 28 days culture in a simple Type-I collagen hydrogel. This approach was chosen in order to provide a biomimetic but otherwise minimally instructive environment which would enable the recapitulation of native cell behaviour, whilst being a high-throughput, reproducible and easily interrogated model for bone formation [9].

Our aims were to distinguish between two hypotheses. Our initial hypothesis, based on previous evidence from foetal chick femur rudiments, was that the cells would respond in a directly proportional way to dynamic pressure waves, i.e. increasing the frequency and magnitude of stimulation from zero would result in a linear increase in bone matrix synthesis [1]. Our competing hypothesis was that this specific hydrostatic stimulus acting on a monoculture of stem cells would need to exceed a threshold level in order to provide enough force to trigger more specific mechanotransduction mechanisms which initiate osteogenic differentiation such as stretch-activated ion channels and ATP release [20, 21].

We subjected the cell-seeded hydrogels to a hydrostatic pressure regime of 280 kPa and 1 Hz for one hour per day over 28 days to simulate the pressure changes experienced in bone interstitial fluid, which we have previously demonstrated has osteogenic effects in a chick foetal femur model [1, 22]. Additional regimes of low pressure (70 kPa) and low frequency (0.05 Hz) were employed, in addition to static, non-cycling high pressure (280 kPa) and static culture at ambient atmospheric pressure (0 kPa). Analysis by non-destructive X-ray microtomography (μCT) at seven-day intervals allowed the progression of mineralisation and changes to the internal hydrogel structural morphometry to be observed, generating numerical data which was supported by end point analysis and quantification of hydrogel calcification [23].

Our objectives were to determine which of our two competing theories (proportional versus threshold responses to hydrostatic pressures) were supported by these data in order to gain a better understanding of mechanosensation in hMSCs and its applications in bone tissue engineering.

## Materials and methods

### Cell culture and collagen hydrogels

Human mesenchymal stem cells were isolated by plastic adherence from a whole bone marrow aspirate (Lonza, Gaithersburg, MD, USA) and cultured to passage 3 in basal DMEM containing 10% foetal calf serum and 1% penicillin–streptomycin (all Gibco, Waltham, MA, USA). MLO-A5 cells were kindly donated by Professor Linda Bonewald of the University of Missouri, Kansas. Cells were trypsinised and incorporated into a collagen hydrogel made from a stock solution of 9.81 mg/ml Type-I rat tail collagen (BD Biosciences, Berkshire, UK) and culture media in 24-well transwell culture inserts (Corning, Deeside, UK). The seeding conditions were 3 × 10^5^ cells per hydrogel, 2.5 mg/ml final collagen concentration with a 300 µl initial volume. After 24 h, the cell-seeded hydrogels were transferred into 24-well plates containing 2 ml fresh osteogenic media (DMEM as above, plus ascorbic acid, 10^−8^ M dexamethasone and 2 mM sodium β-glycerophosphate, all Sigma-Aldrich, Gillingham, UK) and cultured with daily media changes.

### Hydrostatic bioreactor

Cyclic hydrostatic pressures were applied to the 3D hydrogels and 2D cell cultures using a custom-made bioreactor designed and built in collaboration with Instron/Tissue Growth Technologies (Minnetonka, MN, USA) [1]. The bioreactor chamber is a sealed, anodised aluminium vessel accommodating a standard-sized cell culture plate (with the lid removed) allowing for pressure changes to be transferred to the gas phase above the well plate and transduced into the cell culture media. Compressed, recycled incubator air was fed from a continuously-running scroll compressor via a heater (to maintain the temperature of the inlet gasses at 37 °C) and through a system of valve gear into the chamber through a sterile cartridge filter. The gas was removed from the chamber by a vacuum mechanism and directed back into the incubator to be recycled. The operation of the valves and heater was controlled by TGT’s GrowthWorks software, allowing fine control of sinusoidal pressure waveforms from 0.0001 Hz to 2 Hz (or a constantly applied pressure) and between 0 and 280 kPa pressure.

### Bioreactor stimulation regimes

**Fig. 1:**
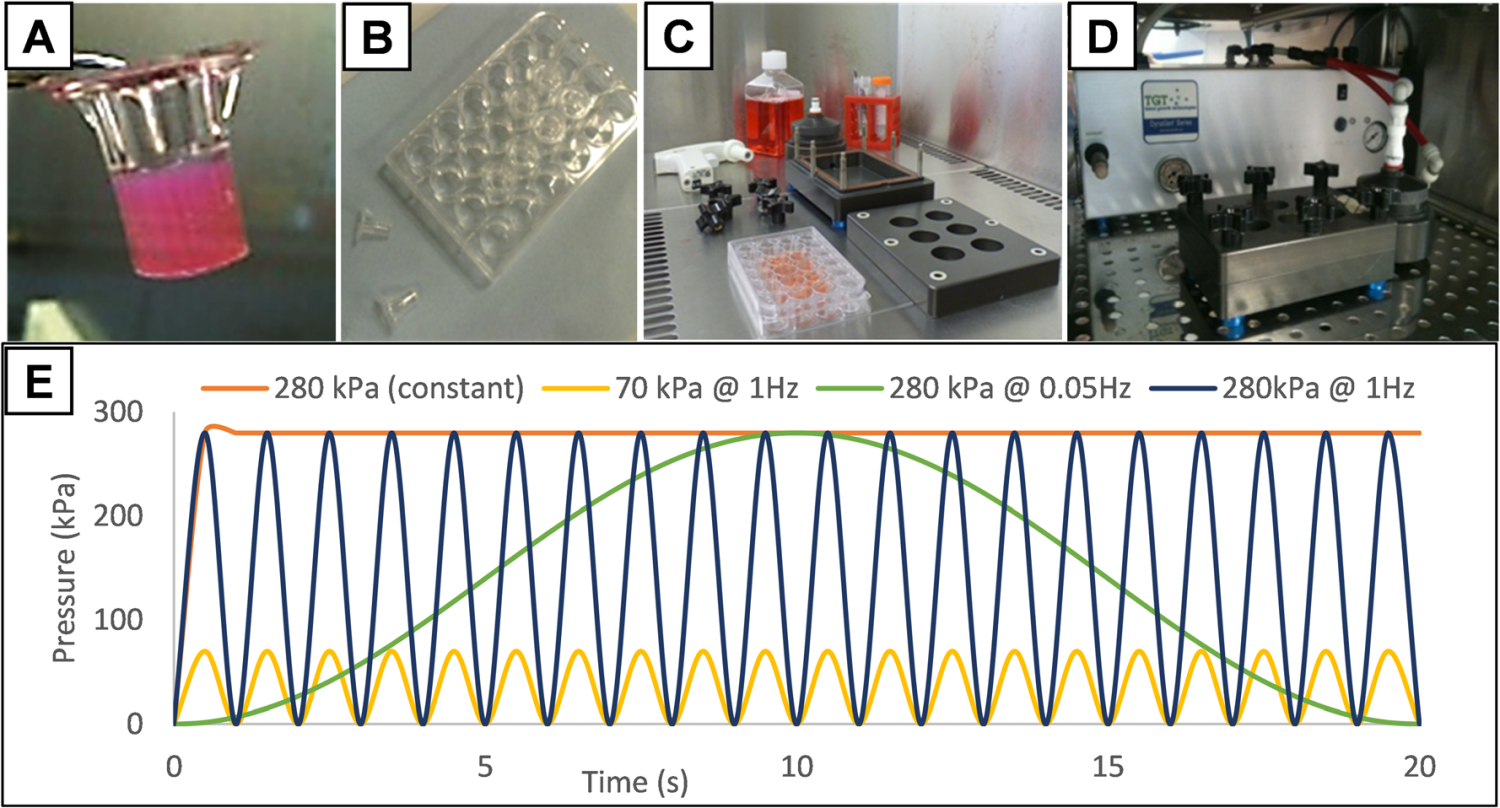
Hydrostatic pressure bioreactor: Experimental set up and loading regimes. **A**, Human MSCs were seeded in collagen hydrogels within transwell insert moulds suspended in a 24-well plate, **B**. 24 h after seeding, the plate containing the suspended hydrogels was placed into the anodised aluminium bioreactor chamber, **C**, which was sealed and connected to the bioreactor hardware, comprising a valve-based pressure regulator which used compressed, recirculated incubator air to apply pressure to the headspace above the culture plate at the required cycling regime, **D**. **E** shows representative traces of the loading regimes employed in this experiment: 0 kPa (static control); 280 kPa constant pressure; 0–70 kPa cycled at 1 Hz; 0–280 kPa cycled at 0.05 Hz; 0–280 kPa cycled at 1 Hz

The hydrogels were cultured for 28 days, with one hour per day in the hydrostatic bioreactor, the remainder of the experimental time period being under standard cell culture conditions in a conventional 37 °C 5% CO_2_ incubator. The pressure regimes applied were: A, No stimulation (static control in incubator); B, 280 kPa constant pressure with no cycling; C, 70 kPa at 1 Hz; D, 280 kPa at 0.05 Hz; E, 280 kPa at 1 Hz (Fig. 1). The hydrogels were cultured in 2 ml osteogenic media, which was removed and replaced daily.

### X-ray microtomography

Analysis of the cell-seeded hydrogels was by X-ray microtomography (μCT) using a Scanco μCT40 (beam energy: 55 kVp, beam intensity: 145 μA, 200 ms integration time, spatial resolution: 10 μm). The hydrogels were removed from culture at 7-day intervals and imaged under sterile conditions using X-ray microtomography (duration < 1 h) and returned to culture. The hydrogel scans were analysed at two density thresholds (50/1000 and 80/1000), firstly to determine the total size (volume) and average density of each hydrogel, and secondly at a higher threshold to determine the volume of the hydrogel that had become mineralised. These thresholds were determined from our previous research on foetal bone development and were conserved throughout these experiments, allowing for longitudinal comparison of hydrogel mineralisation across the investigation.

### Analysis of ECM composition

Five of the six hydrogels from each group were assayed for ECM composition at day 28 of the experiment. The hydrogels were fixed in 4% paraformaldehyde for 20 min, then washed in PBS. The fixed hydrogels were imaged before and after the staining and destaining procedure using a stereoscopic dissecting microscope. Calcium deposition was determined by immersing the hydrogels in a 1% alizarin red solution for 4 h. The hydrogels were then immersed in several changes of water for 48 h to remove unbound dye, then imaged and individually destained in 2 ml 5% cetylpyridinium chloride solution, the absorbance of which was quantified using a spectrophotometer (at 562 nm) to determine the amount of calcium present in the hydrogels (all reagents from Sigma-Aldrich).

### Polyacrylamide gels and surface functionalisation

Polyacrylamide gel substrates were created with different stiffness and functionalised. 250 μl of 0.1 M sodium hydroxide (NaOH) was dispensed onto the surface of a 13 mm glass coverslip (Fisher Scientific, Loughborough, UK) and placed on a hotplate at 70 °C to allow evaporation and formation of an even coating. The coverslips were then coated with 200 μl (3-Aminopropyl)triethoxysilane (Sigma-Aldrich) for 5 min in a fume hood and washed for 30 s under running dH_2_O. The coverslips were then immersed in a 0.5% glutaraldehyde/PBS solution (Sigma-Aldrich) for 30 min and left to dry overnight. Chloro-silinated glass slides were prepared by coating glass slides in 100 μl dichlorodimethylsilane (Sigma-Aldrich) for 5 min in a fume hood, followed by rinsing for 30 s three times with dH_2_O. Solutions of Acrylamide/ Bisacrylamide (Sigma-Aldrich) were prepared in different ratios to generate substrates with stiffnesses of 1, 10 and 40 kPa, degassed in a vacuum desiccator for 15 min, and then mixed with 1/100 10% (w/v) ammonium persulfate (Sigma-Aldrich) by volume and 1/1000 N,N,N′,N′-Tetramethylethylenediamine (Sigma-Aldrich) by volume. 25 μl of the solution was pipetted onto prepared chloro-silinated slides and the amino-coated coverslips placed face down on top. The solutions were allowed to polymerise for 30 min, and then the polyacrylamide coated coverslips were removed from the cholo-silinated slides, transferred to well plates and washed twice in PBS. The coverslips in well plates were then sterilised overnight under UV light in a class II biological safety cabinet. The polyacrylamide substrates were then functionalised by immersion in 200 μg/ml sulfosuccinimidyl 6-(4'-azido-2'-nitrophenylamino) hexanoate (Sulpho-SANPAH) (Thermo-Fisher, Altrincham, UK) in DMSO/dH_2_O, cured under a 365 nm UV light source in a Bio-rad GenX UV chamber for 10 min, rinsed twice in 50 mM HEPES and 0.2 mg/ml Type I collagen solution added (BD Biosciences) and allowed to incubate overnight at 37 °C. The collagen solution was then aspirated and the coverslips washed first in PBS and then in culture media prior to cell seeding.

### Immunofluorescence (actin and YAP)

Monolayer cell cultures were washed briefly in PBS, fixed in 10% neutral-buffered formalin (Fisher Scientific) and permeabilised by adding 0.01% triton-X 100 (Sigma) for 15 min, followed by washing in PBS- 0.1% Tween (Sigma), and then blocking in 1% bovine serum albumin (BSA) (Fisher Scientific) for 1 h followed by two washes in PBS-0.1% Tween (all steps carried out at room temperature). Primary antibodies (anti-human YAP mouse monoclonal, Santa Cruz Bio, Dallas, TX, USA; anti-human osteocalcin mouse monoclonal, R&D systems Minneapolis, MN, USA; anti-human Runx2 goat monoclonal, R&D systems) were made to 2 μg/ml using a solution of 0.1% BSA in PBS with 0.1% v/v tween. The antibody solutions were incubated with the fixed cells overnight at 4 °C, then the cell layers washed twice in PBS-0.1% Tween and incubated with either Alexafloura 488 or 555 (2 μg/ml, 0.1% BSA, 0.1% Tween-20 in PBS) (Abcam, Waltham, MA, USA) in the dark for 1 h at room temperature, followed by two PBS-0.1% tween washes. Cell nuclei were counterstained with 4',6-diamidino-2-phenylindole (DAPI) (Sigma-Aldrich) for 30 min, and F-actin stained with Cytopainter 555 phalloidin (Abcam) for 1 h, with both steps performed at room temperature in the dark. The coverslips were mounted onto microscope slides with Vector shield medium (Vector Laboratories, Newark, CA, USA), and imaged on three fluorescence channels using an Olympus U-TBI 90 laser scanning confocal microscope. Images were acquired using the Fluoview10 software and analysed with Fiji/ImageJ software on a Win64 operating system (http://fiji.sc/Fiji).

### Quantification of staining

Fluorescence images were acquired with identical offset and gain settings for each sample and experiment, and fluorescence intensity analysed in ImageJ to quantify changes in the ratio of nuclear to cytoplasmic concentration of YAP. The nuclear fluorescence intensity values were subtracted from the total cell fluorescence values, and then normalised to the cell area to give a nuclear: cytoplasmic fluorescence intensity ratio per cell.

### Statistical analysis

Numerical data was analysed with GraphPad Prism 8 using a Student’s t-test or one-way analysis of variance (ANOVA) with multiple comparison tests. Significance was set at *p* < 0.05. Data are presented as mean values ± standard deviation.

## Results

**Fig. 2:**
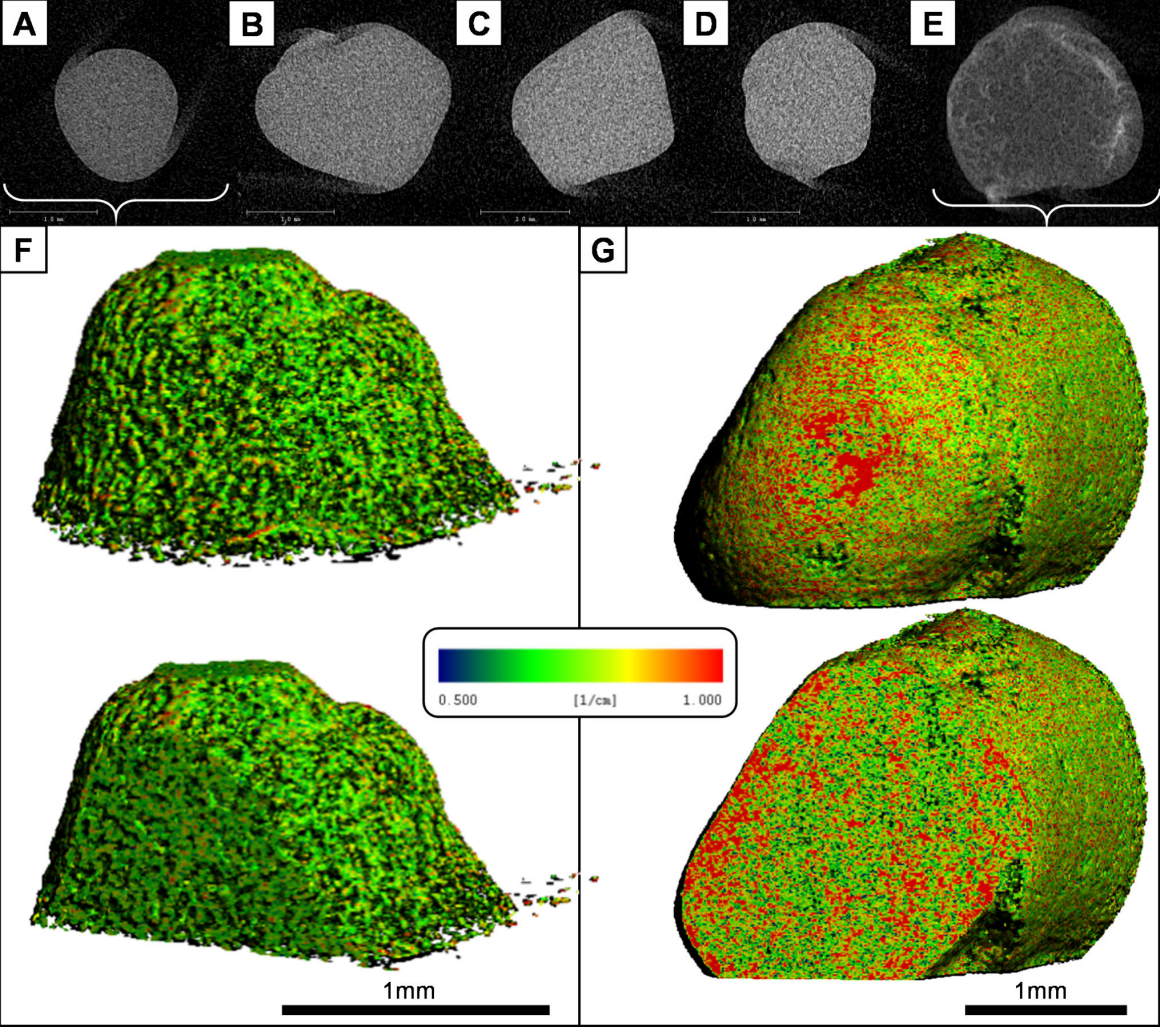
μCT reveals structural changes caused by mineralisation of the hydrogels in response to dynamic hydrostatic pressure. Individual 2D X-ray images of hMSC-seeded hydrogels from all groups: **A**, Static control; **B**, 280 kPa constant for 1 h; **C**, 70 kPa cycling at 1 Hz for 1 h; **D**, 280 kPa cycling at 0.05 Hz for 1 h; **E**, 280 kPa cycling at 1 Hz for 1 h. Significant changes in density distribution only occur in the 280Kpa @ 1 Hz group, with mineralisation being most prevalent in the sub-surface zone of the hydrogel. F & G show false colour reconstructions and cross-sections of representative hydrogels at 28 days, from control (**F**) and stimulated (280 kPa @ 1 Hz, **G**) groups, spectrally shaded at specific density thresholds, such that green voxels represent unmineralised matrix, and red areas show areas of mineral deposition. These images are representative of the sample set, n = 6. Scale bar is 1 mm

Applying a dynamic pressure at 280 kPa at 1 Hz significantly increased the density of the constructs in comparison to controls and the other stimulation regimes (Fig. 2). X-ray microtomography reconstructions revealed the extent of the changes in hydrogel density, with large areas of the construct appearing red (mineralised) in spectrally shaded false colour reconstructions of the constructs, compared to the control group (Fig. 2).

**Fig. 3:**
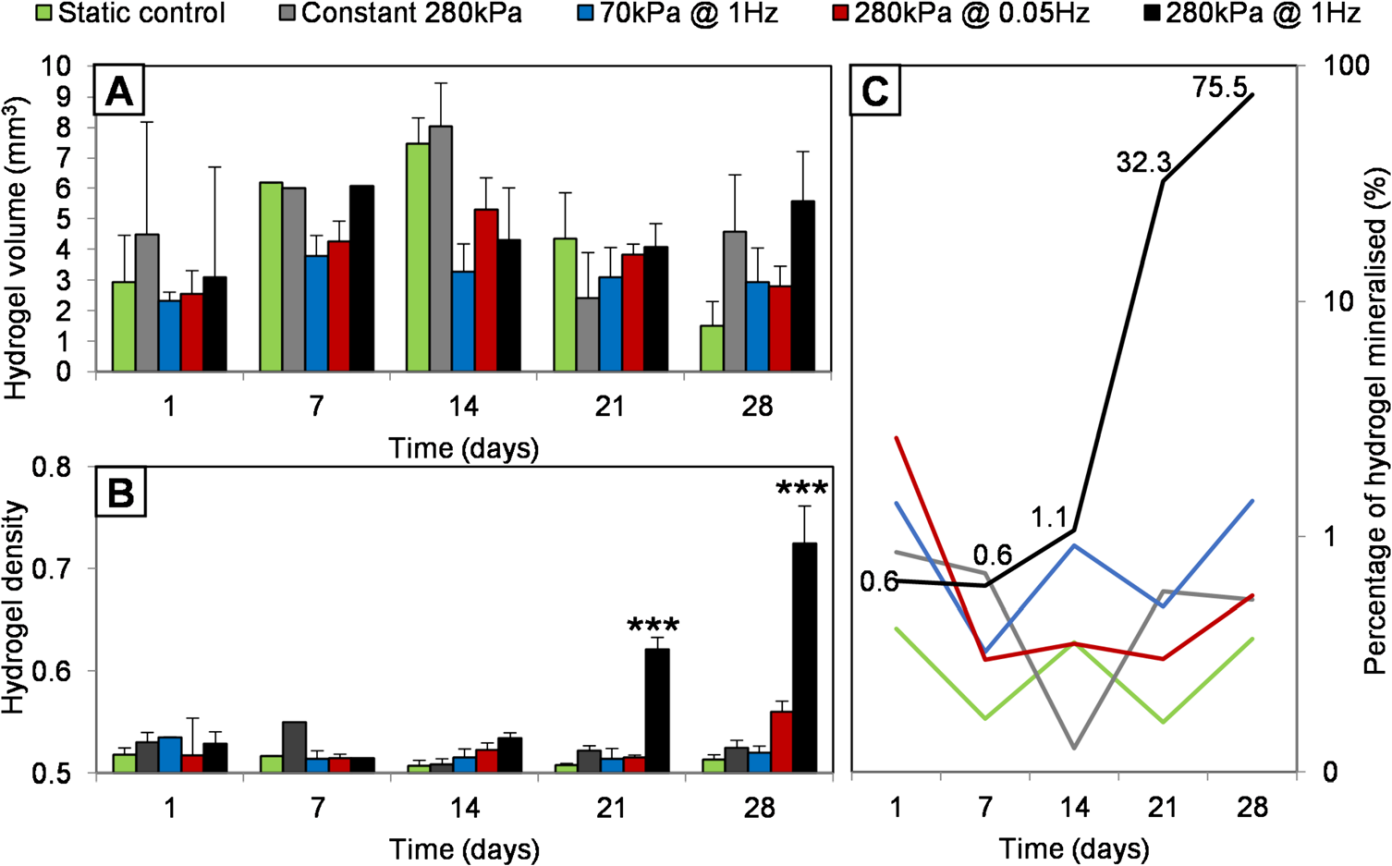
hMSC-seeded hydrogels change in size and density over time in response to dynamic hydrostatic pressure. X-ray microtomography (μCT) analysis of the hydrogels at weekly time points from 24 h after initial seeding (post-contraction, day 1) to 28 days and termination of the experiment, **A**. After the initial contraction, the total volume of the hydrogel constructs increased slightly up to 14 days. **B**, The density of the constructs was evaluated as a measure of mineralised tissue formation and found to increase only in the group receiving 280 kPa cyclic pressure at 1 Hz. **C**, Comparing the volume of the construct above an established mineralisation threshold (> 80) to the total construct volume (> 50) at each time point allowed the progress of mineralision to be tracked as a percentage of the whole volume mineralised. Only the group receiving 280 kPa cyclic stimulation at 1 Hz showed significantly more than 1% mineralisation (P < 0.001), and progression of mineralisation was rapid after 14 days. Error bars show standard error of the mean, n = 6; *** = *p* < 0.001

Total hydrogel volume increased in all samples to a peak at ~ 14 days, followed by a general decrease in volume as the constructs appeared to contract (Fig. 3A). The relative density of the hydrogels cultured with 280 kPa at 1 Hz showed significant increases in relative density from day 21 onwards, compared to other groups and the control (Fig. 3B). The volume of the hydrogel at different density thresholds was determined by defining mineralising matrix as any material over a threshold of 80, and total hydrogel volume as material over 50. It was possible to quantify the volume of the hydrogel that was becoming mineralised by comparing these values (V^80^/V^50^) at each 7-day time point. Whilst neither the control or other stimulation groups showed significant mineralisation (less than 2% mineralised at day 28), the group receiving 280 kPa at 1 Hz was on average 75% mineralised by the end of the experiment (Fig. 3C).

**Fig. 4:**
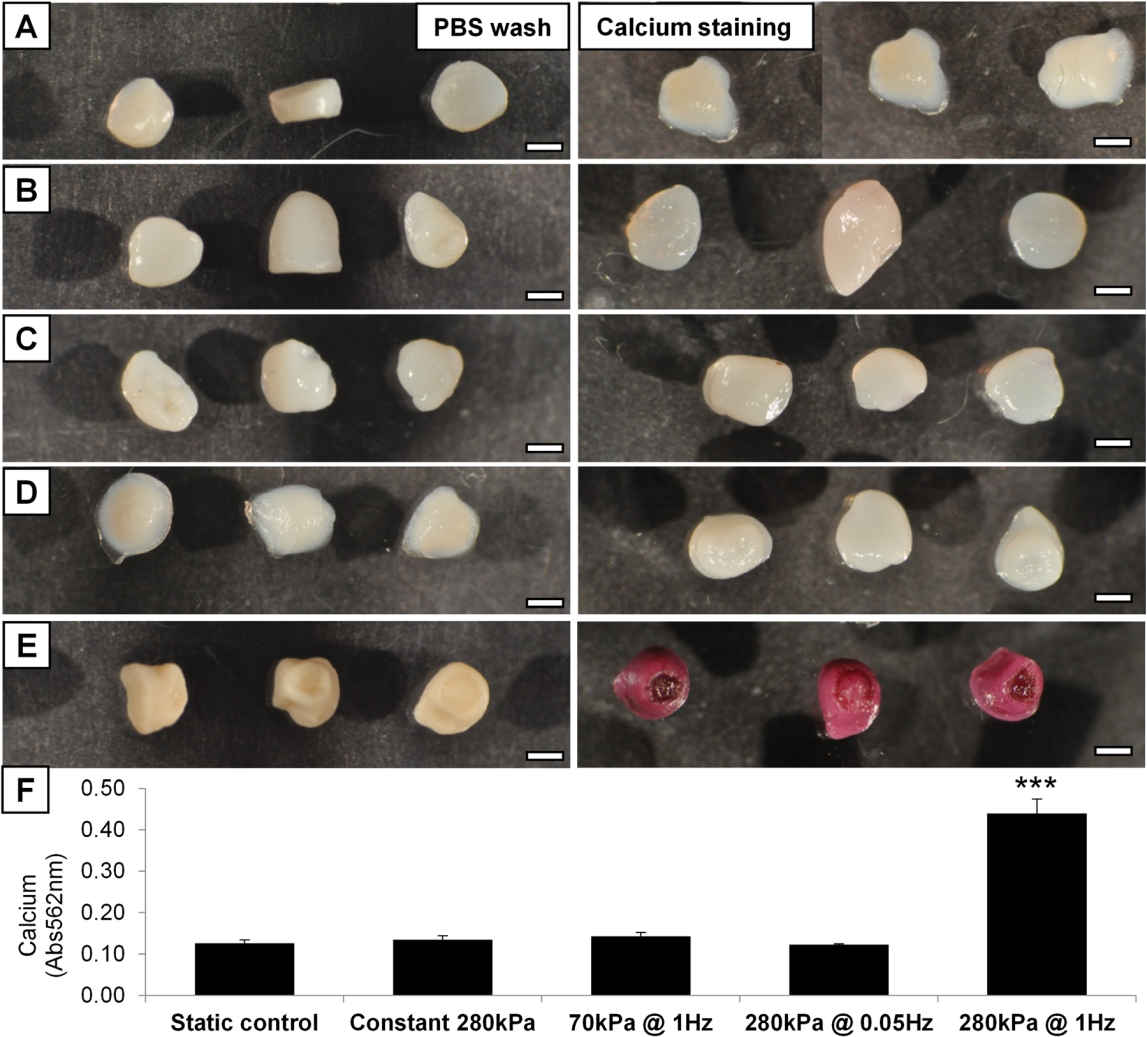
Calcification of the hMSC-seeded hydrogels was observed and quantified. Hydrogels from all groups were removed from culture at 28 days, washed in PBS (left panel) and stained for calcium with Alizarin Red S solution (right panel). Images show **A**, Static control; **B**, 280 kPa constant for 1 h; **C**, 70 kPa cycling at 1 Hz for 1 h; **D**, 280 kPa cycling at 0.05 Hz for 1 h; **E**, 280 kPa cycling at 1 Hz for 1 h. The cell-seeded hydrogels directly after PBS wash showed differences in opacity *vs* translucence under the different treatments after 28 days in culture. After staining for calcium deposits with alizarin red only the 280 kPa@1 Hz group had significant staining. Visual analysis was supported by quantification of calcification using spectrophotometric analysis of the destain solution (5% cetylpyridinium chloride) at 562 nm revealing a significant increase in the samples stimulated at 280 kPa @1 Hz. Error bars show standard error of the mean, n = 5; *** = *p* < 0.001. Scale bar is 1 mm

Differences in the hydrogels were also evident macroscopically, as the 280 kPa 1 Hz stimulated samples were visibly more opaque than the translucent control hydrogels indicating an accumulation of extracellular material (Fig. 4 & S1). The hydrogel constructs were stained with alizarin red for calcium, followed by washing in a mild destaining detergent which revealed that whilst the control and other groups showed minimal mineralisation in the matrix, the 280 kPa 1 Hz stimulated group was substantially stained for deposited calcium (Fig. 4). Analysis of the cetylpyridinium chloride destain solutions showed that the total amount of calcium in these hydrogels was more than four-fold higher than controls, with no significant calcification an any other stimulated group (Fig. 4E).

**Fig. 5:**
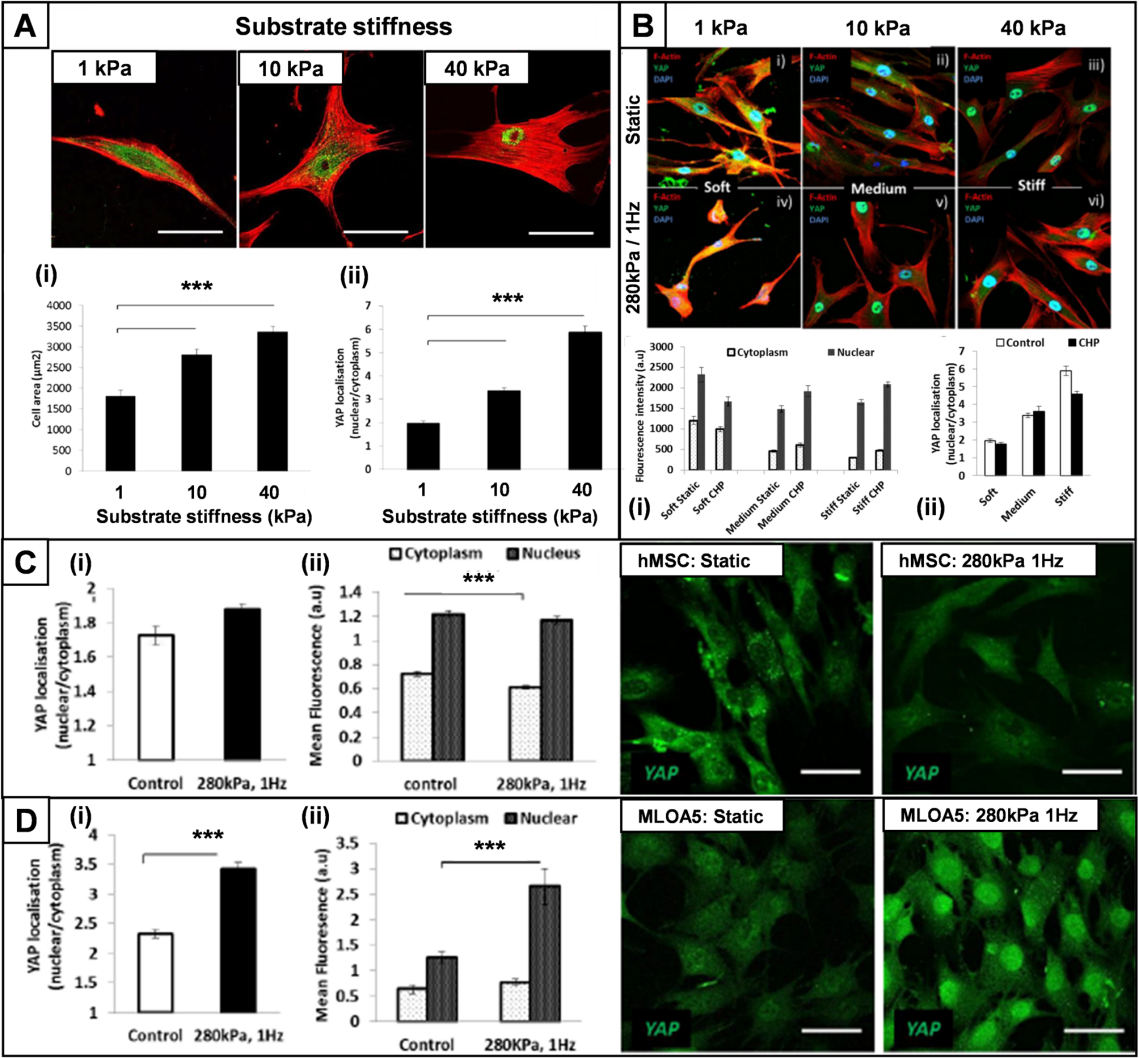
Mechanotransduction of hydrostatic pressure is not initially via nuclear translocation of YAP in undifferentiated hMSCs. **A**, Immunofluorescent staining showing F-actin (red) and YAP expression (green) in hMSCs seeded on polyacrylamide substrates of differing stiffness. Substrate stiffness correlated with increased cell spreading (Aii) and increased nuclear translocation of YAP under static conditions prior to hydrostatic pressure. **B**, No significant differences were observed in total cytoplasmic YAP expression or nuclear translocation in response to cyclic hydrostatic pressure applied at 280 kPa, 1 Hz for 1 h as either absolute values in the cytoplasm and nucleus (Bi), or as a ratio of nuclear/cytoplasmic localisation (Bii). **C**, Repeating the stimulation experiment with hMSC on tissue culture plastic again showed no significant increase in nuclear translocation, but indicated a small but significant decrease in cytoplasmic YAP levels (Cii), (n = 40). **D**, In contrast, the mature osteoblast cell line MLOA5 showed significant nuclear translocation of YAP in response to 1 h stimulation with cyclic hydrostatic pressure at 280 kPa and 1 Hz. Ciii and Diii are representative confocal images showing YAP expression under standardised fluorescence settings for hMSC and MLOA5 cells respectively, under control conditions (static culture) and following 60 min of hydrostatic pressure applied at 280 kPa at 1 Hz. Error bars show standard error of the mean, n = 40, *** = *p* < 0.001. Scale bar is 50 μm

A range of functionalised polyacrylamide surfaces were manufactured to determine the relationship between cytoskeletal spreading area and nuclear translocation of YAP in response to substrate stiffness. MSCs seeded on soft (1 kPa) substrates had a spindle-like morphology with low cytoskeletal spread, and both cytoskeletal spreading area and nuclear YAP localisation increased proportionally with substrate stiffness from 1 to 40 kPa (Fig. 5A). There was no significant change in YAP nuclear localisation when cyclic hydrostatic pressure was applied at 280 kPa and 1 Hz for 1 h on any substrate, although the proportional relationship with stiffness and YAP nuclear/cytoplasmic ratio was maintained (Fig. 5B). Repeating the stimulation experiment with hMSC on tissue culture plastic (~ 10^7^ kPa) again showed no significant increase in nuclear translocation, but indicated a small but significant decrease in cytoplasmic YAP levels (Fig. 5C). In contrast, the mature osteoblast cell line MLO-A5 showed significant nuclear translocation of YAP in response to 1 h stimulation with cyclic hydrostatic pressure at 280 kPa (Fig. 5D).

## Discussion

Controlling the behaviour of cell and tissue cultures via their mechanical environment is a promising approach for unlocking the potential of tissue engineering at all scales [24–26]. In this investigation we set out to define the magnitude and frequency of hydrostatic pressure needed to stimulate human MSCs to differentiate and mineralise their extracellular matrix using a bioreactor. Our broad objectives were to determine if the osteogenic response of hMSCs to cyclic hydrostatic pressure was directly proportional to the frequency and magnitude of stimulation, or if an activation threshold was required to trigger a cell response.

Dynamic pressures have been shown to elicit transient transcriptomic changes in hMSCs at pressures as low as 10 kPa, with significant osteogenesis occurring at 300 kPa [27]. In addition to dynamic environmental pressure, the stiffness of the substrate to which the cells attach is known to be critical in determining how hydrostatic pressure is transduced by cells, with stiffer substrates enabling increased mechanotransduction via the cytoskeleton [28]. In the 3D collagen hydrogel model we used here, 70 kPa was found to be below the critical pressure threshold needed to trigger full osteogenic differentiation and mineralisation, whereas 280 kPa was shown to be sufficient to drive substantial and significant *in vitro* bone formation. We discovered that the minimum frequency of stimulation (or possibly the total number of cycles) is also important, with low frequencies of 0.05 Hz for 1 h having no effect on osteogenesis even at 280 kPa pressure. This corresponds to *in vivo* studies suggesting a minimum stimulation frequency of 0.5 Hz is required to elicit an osteogenic response in adult bone and compares reasonably well to the loading frequency generated by normal gait locomotion, i.e. 30–60 walking/running steps per minute [27, 29].

Our previous research using chick foetal femurs as a model for endochondral bone formation revealed that hydrostatic pressure has a profound effect on the progress of mineralisation in the developing diaphyseal bone collar [1]. In those developing tissues, dynamic pressure caused an increase in both the amount of bone formed and its density (a measure of its adaptation to a mechanical environment). We also found that the frequency of stimulation was directly proportional to the increase in bone density, whilst femurs isolated from older foetuses (i.e., with more mature bone cells) responded more strongly to dynamic pressure than immature tissues containing a higher proportion of stem, progenitor and chondroblastic anlage cells. Together with the results from this current investigation, there is growing evidence to suggest that cells at different developmental stages and occupying different biological niches likely respond to hydrostatic pressure in dissimilar ways, leading to speculation that skeletal cells can adapt their mechanosensitivity thresholds and transduction mechanisms over the life course and as an adaption to their environment. These mechanisms may also include elements of cellular memory, which are of interest in a range of cell-based therapies, musculoskeletal ageing and clinical medicine [30, 31].

Many mechanotransduction mechanisms have been described for hydrostatic pressure, and it is difficult to determine within one study which combinations of these pathways are activated [32, 33]. The Hippo pathway has emerged as a mechanism of interest in mechanobiology due to its potential as bioengineering control point and drug target [34]. In order to assess the activity of YAP/TAZ in our bioreactor, we developed 2D substrates representing substrates of different stiffness to allow comparison with our 3D hydrogels and with the wider literature. Collagen hydrogels have a stiffness of between 1 and 10 kPa, and we considered it plausible to assume that stiffer, mineralising extracellular matrices of up to 40 kPa were being generated over the 28 days in our 3D experiments. Published work on substrate stiffness from Dupont et al., used these same stiffnesses in their work on MSC YAP/TAZ nuclear translocation in mechanotransduction [17]. Our objectives were to identify commonalities in MSC mechanotransduction between published work and our own 2D and 3D cultures to enable further investigation of the Hippo pathway as the mechanism by which MSC sense and respond to hydrostatic pressure. Our hypothesis was that MSCs cultured on soft, hydrogel-like substrates (1 and 10 kPa) and exposed to cyclic hydrostatic pressure would have increased nuclear YAP, which would enable us to subsequently utilises the molecular tools developed for interrogating the Hippo pathway *in vitro* for our research and enable some translational applications of this work.

We compared the response of mesenchymal stem cells with mature murine MLO-A5 osteoblast (pre-osteocyte) cells to hydrostatic pressure and on substrates of increasing stiffness. Hydrostatic pressure triggered the translocation of YAP to the nucleus in MLO-A5 murine mature osteoblast (pre-osteocyte) cells and was associated with f-actin stress fibre formation. In contrast, nuclear accumulation of YAP did not occur in human MSCs as a result of hydrostatic pressure, but only in response to increasing substrate stiffness (as has been well described by several other researchers) [17]. We therefore cannot confirm at this point that Hippo YAP/TAZ is responsible for mechanotransduction of hydrostatic pressure in our 3D model.

YAP/TAZ has been shown to transduce dynamic hydrostatic pressures in other mature cell types, e.g. HEK293, so it is presently unclear if underlying substrate stiffness, species differences, or changes in mechanotransduction mechanisms for hydrostatic pressure between undifferentiated stem cells and mature osteoblasts best explain our data [35, 36]. Nuclear translocation of YAP has been shown to depend on f-actin stress fibre formation, which is widely reported in osteoblasts undergoing mechanical loading and hMSCs responding to substrate stiffness. These data comparing undifferentiated hMSC with mature osteocyte cell lines suggest that the mechanosensation mechanism changes as the cells become differentiated, as has been demonstrated for embryonic stem cells which increase in stiffness as they differentiate [37]. Sensitivity of cells to hydrostatic pressure may therefore be regulated different mechanisms during the differentiation process, in which cytoskeletal changes enable an increased sensitivity to hydrostatic pressure which can be transduced via the Hippo pathway. These changes may therefore reinforce the phenotypic shift and help facilitate lineage commitment. Differentiated osteoblasts may thus become more sensitive to hydrostatic pressure as they mature into osteocytes and adapt their mechanosensation to respond to the active bone microenvironment.

Engineered living grafts consisting of a cell-seeded biomaterial are becoming an established paradigm for the clinical reconstruction of lost, diseased and damaged bone. Preconditioning regimes in which the cells are cultured under an appropriate level of mechanical stress prior to implantation may improve the functional quality and rate of manufacturing these grafts by maximising the osteogenic potential of the cells. This research indicates that cyclic hydrostatic pressure is a relatively precise tool for controlling stem cell osteogenesis *in vitro* for tissue engineering. Previous studies in which this bioreactor was used to stimulate osteogenesis in ex vivo cultured chick femurs revealed increases in osteochondral gene expression (including COL2, osteopontin and CD44) in response to cyclic hydrostatic pressure, indicating that mechanical stimuli were being transduced into transcriptomic responses and ultimately into cell-mediated changes in bone development and mineralisation [1]. In developing femora, a diverse population of cells at differing levels of maturation are present, and so our rationale for this initial investigation into cell-seeded hydrogels was to address the sensitivity of a monoculture of hMSCs to different magnitudes and frequencies of compressive mechanical force. Both the initial stimulus detection mechanism and subsequent intracellular signal transduction are likely to involve multiple receptors, molecular deformations and the activation of stretch-activated ion channels, and the downstream signals inevitably propagate via several interactive pathways to elicit changes in transcription [4, 9, 12, 15].

Our aim in this initial investigation was to address the question of hMSC sensitivity to different magnitudes and frequencies of compressive mechanical force. Now that we have determined that these hMSC require specific levels of force to differentiate we can proceed towards understanding the cell molecular biology underpinning these thresholds.

## Supplementary Information

Below is the link to the electronic supplementary material.

Supplementary file1 (DOCX 1760 kb)

## Data Availability

The original contributions presented in the study are included in the article; further inquiries can be directed to the corresponding authors.
