# Supplementary material for: Determining Which Hydrostatic Pressure Regimes Promote Osteogenesis in Human Mesenchymal Stem Cells
Source: Tissue Eng Regen Med. 2024 Aug 27;21(8):1141–51. doi: 10.1007/s13770-024-00666-w (PMC11589021; doi:10.1007/s13770-024-00666-w)
Supplement: Supplementary file 1 — Supplementary file1 (DOCX 1760 kb) [file 13770_2024_666_MOESM1_ESM.docx]

Fig. S1. Full panel of hydrogels following 28 days culture. Hydrogels from all groups were removed from culture at 28 days, washed in PBS and imaged using a dissecting microscope. Images show A, Static control; B, 280 kPa constant for 1 hour; C, 70 kPa cycling at 1Hz for 1 hour; D, 280 kPa cycling at 0.05 Hz for 1 hour; E, 280 kPa cycling at 1Hz for 1 hour. The cell-seeded hydrogels directly after PBS wash showed differences in opacity vs translucence under the different treatments after 28 days in culture


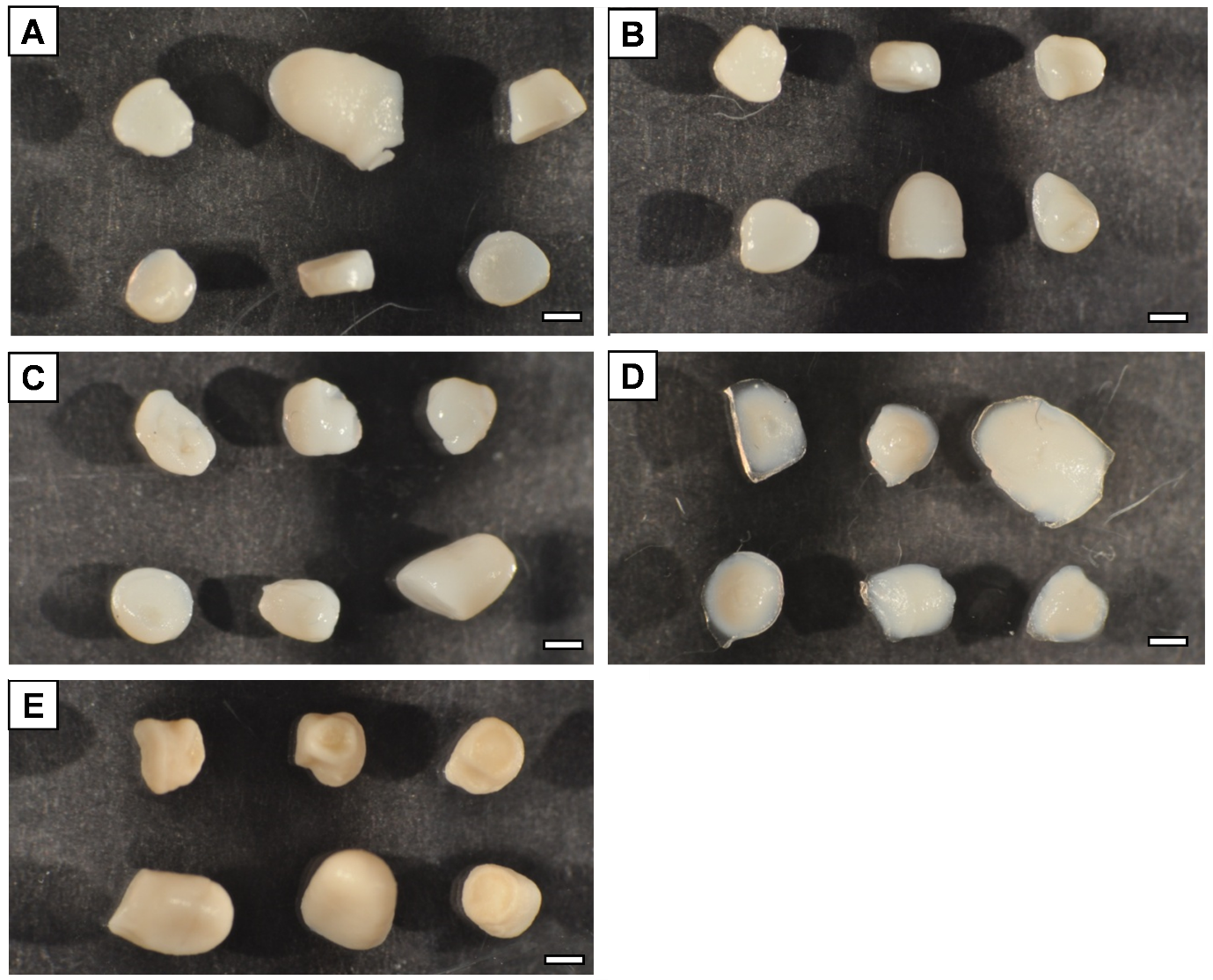
S
